# Association between physical function and long-term care in community-dwelling older and oldest people: the SONIC study

**DOI:** 10.1186/s12199-020-00884-3

**Published:** 2020-09-01

**Authors:** Werayuth Srithumsuk, Mai Kabayama, Kayo Godai, Nonglak Klinpudtan, Ken Sugimoto, Hiroshi Akasaka, Yoichi Takami, Yasushi Takeya, Koichi Yamamoto, Saori Yasumoto, Yasuyuki Gondo, Yasumichi Arai, Yukie Masui, Tatsuro Ishizaki, Hiroshi Shimokata, Hiromi Rakugi, Kei Kamide

**Affiliations:** 1 Department of Health Promotion System Sciences, Division of Health Sciences, Graduate School of Medicine, Osaka University, Osaka, Japan; 2 Department of Geriatric and General Medicine, Graduate School of Medicine, Osaka University, Osaka, Japan; 3 Department of Clinical Thanatology and Geriatric Behavioral Science, Graduate School of Human Sciences, Osaka University, Osaka, Japan; 4 Center for Supercentenarian Medical Research, Keio University School of Medicine, Tokyo, Japan; 5 Tokyo Metropolitan Geriatric Hospital and Institute of Gerontology, Tokyo, Japan; 6 Graduate School of Nutritional Sciences, Nagoya University of Arts and Sciences, Aichi, Japan

**Keywords:** Frailty, Grip strength, Long-term care, Older people, Walking speed

## Abstract

**Background:**

Preventing the need for long-term care (LTC) by identifying physical function risk factors are important to decrease the LTC burden. The objective of this study was to investigate whether grip strength and/or walking speed, which are components of the frailty definition, are associated with LTC in community-dwelling older and oldest people.

**Methods:**

The participants were 1098 community-dwelling older and oldest people who had not received LTC at the baseline. The endpoint was receiving LTC after the baseline survey. The independent variables were grip strength and walking speed, and participants were divided into two groups based on these variables. The confounding factors were age, sex, the Japanese version of the Montreal Cognitive Assessment (MoCA-J), hypertension, diabetes mellitus, stroke, joint diseases, living alone, body mass index, and serum albumin. We calculated the hazard ratio of receiving LTC using the Cox proportional hazard model.

**Results:**

Among the 1098 participants, 107 (9.7%) newly received LTC during the follow-up. Regarding the physical function, only slow walking speed was significantly correlated with LTC after adjusting for all confounding factors except the MoCA-J score (HR = 1.74, 95% CI = 1.10–2.75, *P* = .018). However, slow walking speed was still a risk factor for LTC after adjusting for the MoCA-J score and other confounding factors (HR = 1.64, 95% CI = 1.03–2.60, *P* = .037).

**Conclusions:**

The findings from this study may contribute to a better understanding of slow walking speed as a factor related to LTC, which might be a criterion for disability prevention and could serve as an outcome measure for physical function in older people.

## Background

In 2000, Japan developed a system of long-term care (LTC) that rendered LTC a universal entitlement for every Japanese citizen aged 65 or older [1]. As a rapid increase in the users of LTC is leading to financial constraints, the Japanese government revised the LTC system in 2006 to concentrate on prevention strategies for those with lower-level needs and those at risk of requiring LTC in the future [2], to minimize these problems. However, previous studies showed that the following are predictive factors for certification to receive LTC: advanced age, impaired walking ability, deterioration in instrumental activities of daily living (IADL), cognitive impairment, treatment of underlying diseases, number of medications, history of falls, loss of excretory ability, functional inactivity, low social activity, poor self-reported health, not able to work, and depression [3–5]. Among such factors, progressive deterioration in physical function and activities of daily living are major health concerns of older people [6]. Therefore, screening for an impaired physical function is very important in the early identification of health risks among older people.

Fried et al. [7] proposed the phenotype model, which measures frailty using the components of the Cardiovascular Health Study (CHS) to describe the age-related decline in physical capacities based on the following criteria: (1) unintentional weight loss, (2) self-reported exhaustion, (3) low grip strength, (4) low physical activity, and (5) slow walking speed. According to this model, the presence of three or more indicates frailty with regard to Japan; Satake et al. [8] advocated a Japanese version of CHS (J-CHS) criteria, which can also identify frailty. The J-CHS’s component and criteria are similar to the original phenotype model. Previous studies of frailty and adverse outcomes examined falls, hospitalizations, disability, death, and admission to LTC facilities [7, 9]. However, no such study has tracked community-dwelling older people developing a need for LTC under home care or community-based care. In a study by Lee et al. [10], it was demonstrated that the combined use of grip strength and walking speed was accurate, precise, specific, and more sensitive for the Fried frailty phenotype than other possible combinations and suggested that these measures could be applied in a primary care setting. More significantly, grip strength and walking speed measurements do not require special equipment, they are not time-consuming or intrusive, and they are helpful in making decisions about interventions and care allocation [11].

To our knowledge, no studies have determined the association of physical function with the incidence of the certified need of care in the national LTC system using handgrip strength and walking speed measurements in older and oldest Japanese people living in the community because in previous studies, data were collected based on self-reports [3, 4] and the follow-up period was between 3 and 4 years [3–5]. Therefore, to prevent older people from showing a declining function in the future, provide them with efficient and effective care, and delay its clinical consequences, investigating the relationship between physical function using grip strength and walking speed as indicators of the need for LTC is useful for community-dwelling older people. Moreover, longitudinal studies can provide information on changes in physical function in older people, such as transitions, trajectories, and factors associated with LTC. Consequently, the objective of this study was to investigate whether grip strength and/or walking speed, which are components of the frailty definition, are associated as physical function indicators with LTC in community-dwelling older and oldest people.

## Methods

### Participants

The study participants were recruited from the Septuagenarians, Octogenarians, Nonagenarians Investigation with Centenarians (SONIC) study, a longitudinal analysis involving the collection of data in the community at the baseline and follow-up every 3 years of a prospective cohort study ongoing since 2010 [12].

**Fig. 1:**
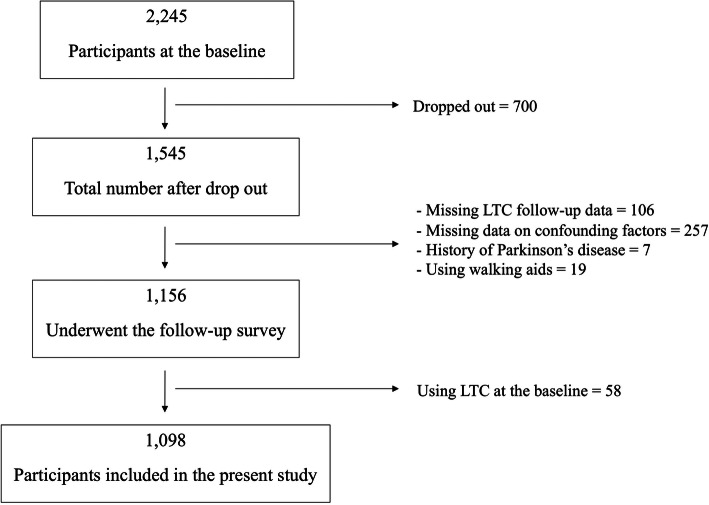
Participants included in the study

The inclusion criteria of this study were as follows: (1) they were free of LTC needs at the baseline, (2) having LTC data at the baseline and follow-up, (3) no missing data for each potential confounding factor, (4) without a history of Parkinson’s disease, and (5) free of the need for walking aids at the baseline. All data were collected at the baseline (2010–2012), while LTC data were collected at the baseline and during follow-up (2013–2018). About 69.63% of the participants were followed up every 3 years (wave 1–wave 3), meaning that participants aged 70 years were followed for 8 years while those aged 80 and 90 years were followed for 7 and 6 years, respectively. The follow-up for the remainder varied from 3 to 8 years. At the baseline, a total of 2245 participants in all age groups (69–71 years old = 1000, 79–81 years old = 973, and 89-91 years old = 272) were included, but only 1098 participants met the inclusion criteria (Fig. 1).

### The LTC assessment

Full details regarding the LTC system in Japan were described previously [1]. The care need levels for LTC services are categorized into the following 7 levels: support need levels 1 and 2 and care need levels 1–5. The higher the level of care need, the more severe the conditions, requiring higher levels of care and services to avoid functional limitations and promote independent living. We identified participants who were receiving some level of LTC services as having received LTC.

### Grip Strength

The participants’ grip strength was performed by trained physical therapists. The dominant grip strength was measured using the Smedley hand dynamometer (Model YD-100; Yagami Ltd., Tokyo, Japan). Measurements were taken in a sitting posture twice, with arms resting against the body. In the analysis, the average of the first and second measurements was used. Weak grip strength was defined as < 26 kg for males and < 18 kg for females [8].

### Walking speed

The participants’ walking speed was implemented by trained physical therapists. The time taken to walk along a 2.44-m course was measured on a walking speed scale. Another measurement was obtained using a digital timer, and the two measurements were combined to give the average walking speed. Slow walking speed was defined according to a cutoff < 1.0 m/s [8].

### Potential confounding factors

Potential confounding factors taken into consideration in the study included age and sex. Living alone was noted in the two groups (living alone and living together with others). A blood sample was obtained, and biochemical tests in grams/deciliter units determined serum albumin levels.

History of stroke and joint diseases was classified in accordance with their yes/no responses. Body mass index (BMI) was measured and formulated as the weight in kilograms divided by the square of the height in meters (kg/m^2^).

Hypertension (HT) was diagnosed in accordance with the 2019 guideline of the Japanese Society of Hypertension [13], characterized by systolic blood pressure (BP) ≥ 140 mmHg and diastolic BP ≥ 90 mmHg or antihypertensive drug use.

Diabetes mellitus (DM) was defined as fasting blood glucose ≥ 126 mg/dL, casual blood glucose ≥ 200 mg/dL, hemoglobin A1c ≥ 6.5%, or antidiabetic drug use by the Japan Diabetes Society [14].

The cognitive function assessment of the participants’ was performed by trained psychologists using the Japanese version of the Montreal Cognitive Assessment (MoCA-J) [15]. The total scores of MoCA-J (0–30 points) were used for assessment of the cognitive function. A higher score indicated greater cognitive function.

### Statistical analysis

The outcome event was the need for LTC. Cases were censored during the follow-up period. First, we carried out a descriptive analysis of the samples; Pearson’s chi-square or Fisher’s exact test for categorical variables and the independent *t* test for continuous variables were used to compare baseline characteristics between groups who had received and not received LTC.

Cox proportional hazard regression analyses were performed using the following 3 models to estimate hazard ratios (HRs) and 95% confidence intervals (95% CI). Model 1 was constructed using the grip strength and walking speed as independent variables. Model 2 included age, sex, HT, DM, stroke, joint diseases, living alone, BMI, and serum albumin as independent variables in addition to grip strength and walking speed. Model 3 included the MoCA-J score in addition to model 2, which was called the adjusted model. Moreover, cox proportional hazard regression analyses were also implemented following stratification by age. *P* values of < 0.05 were considered significant. These statistical analyses were carried out with SPSS Statistics 26.0 (IBM Japan, Tokyo, Japan).

## Results

In the baseline survey, 2245 participants were recruited, but only 1098 (48.91%) had not received LTC at the baseline and met the inclusion criteria, as shown in Fig. 1. After a follow-up of the LTC status, 107 (9.7%) participants had used LTC services. When the participants were grouped based on receiving LTC after follow-up, the independent physical function was also significant based on a weak grip strength on comparing those who received and those who did not receive LTC (53.3 vs. 35.7%, respectively; *P* < .001). Similarly, the slow walking speed in those who received LTC was also assigned a higher percentage than in those not receiving LTC (75.7 vs. 58.2%, respectively; *P* < .001) (Table 1).

**Table 1:** Comparison of baseline characteristics between subjects in non-received LTC and those received LTC groups during follow-up (*n* = 1,098)

Characteristics	Total ***N*** (%)	Non-received LTC (***n*** = 991; 90.3%)	Received LTC (***n*** = 107; 9.7%)	***P*** value
**Grip strength**^a^				
Normal	687 (62.6)	637 (64.3)	50 (46.7)	< .001^d^
Male	389 (56.6)	367 (57.6)	22 (44.0)	.075^d^
Female	298 (43.4)	270 (42.4)	28 (56.0)	
Weak	411 (37.4)	354 (35.7)	57 (53.3)	
Male	152 (37.0)	135 (38.1)	17 (29.8)	.241^d^
Female	259 (63.0)	219 (61.9)	40 (70.2)	
**Walking speed**^b^				
Normal	440 (40.1)	414 (41.8)	26 (24.3)	< .001^d^
Slow	658 (59.9)	577 (58.2)	81 (75.7)	
**MoCA-J score** (Mean ± SD)	23.00 ± 3.44	23.17 ± 3.35	21.44 ± 3.84	< .001
**Age**				
70 y/o	523 (47.6)	508 (51.3)	15 (14.0)	< .001^c^
80 y/o	531 (48.4)	452 (45.6)	79 (73.8)	
90 y/o	44 (4.0)	31 (3.1)	13 (12.1)	
**Sex**				
Male	541 (49.3)	502 (50.7)	39 (36.4)	.006^d^
Female	557 (50.7)	489 (49.3)	68 (63.6)	
**HT**				
No	299 (27.2)	278 (28.1)	21 (19.6)	.068^d^
Yes	799 (72.8)	713 (71.9)	86 (80.4)	
**DM**				
No	931 (84.8)	843 (85.1)	88 (82.2)	.440^c^
Yes	167 (15.2)	148 (14.9)	19 (17.8)	
**Stroke**				
No	1043 (95.0)	943 (95.2)	100 (93.5)	.444^c^
Yes	55 (5.0)	48 (4.8)	7 (6.5)	
**Joint diseases**				
No	824 (75.0)	756 (76.3)	68 (63.6)	.006^d^
Yes	274 (25.0)	235 (23.7)	39 (36.4)	
**Living alone**				
No	909 (82.8)	832 (84.0)	77 (72.0)	.002^c^
Yes	189 (17.2)	159 (16.0)	30 (28.0)	
**BMI** (Mean ± SD)	22.73 ± 2.89	22.75 ± 2.88	22.50 ± 3.00	.386
**Serum albumin** (Mean ± SD)	4.37 ± 0.27	4.38 ± 0.27	4.30 ± 0.27	.005

Independent *t* test for continuous variable

*BMI* body mass index, *DM* diabetes mellitus, *HT* hypertension, *LTC* long-term care, *MoCA-J* The Japanese version of the Montreal Cognitive Assessment, *SD* standard deviation, *y/o* years old

^a^Weakness was defined as a maximum grip strength < 26 kg in males and < 18 kg in females

^b^Slowness was defined as a gait speed < 1.0 m/s.

^c^*P* values from Pearson’s chi-square test

^d^*P* values from Fisher’s exact test

**Fig. 2:**
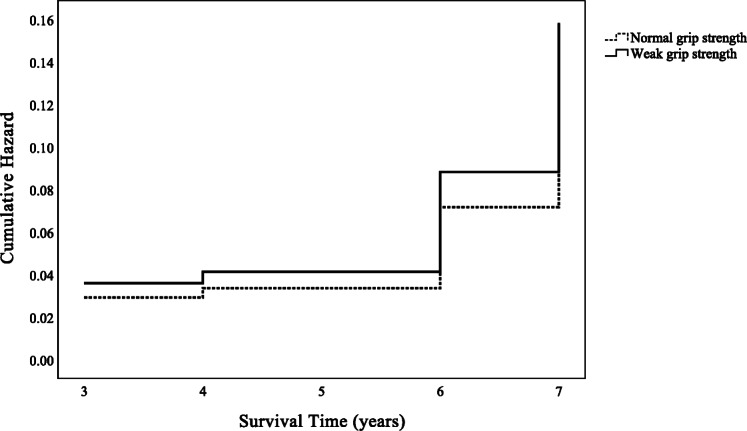
Cumulative hazard of grip strength on long-term care

**Fig. 3:**
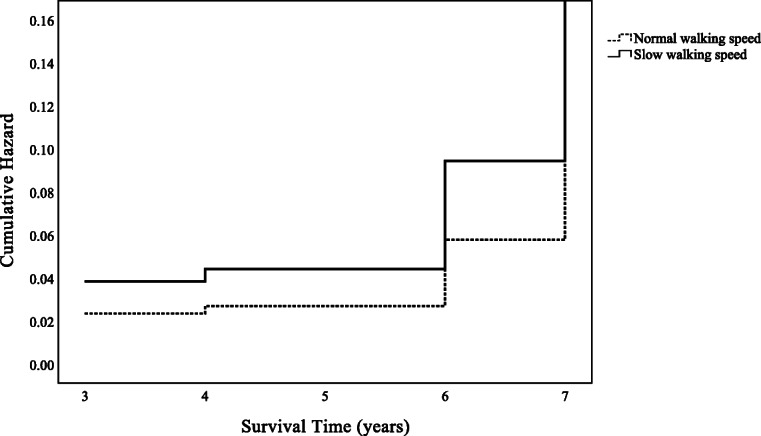
Cumulative hazard of walking speed on long-term care

Table 2 shows findings from the Cox proportional hazard regression analyses. The analysis of independent physical function variables showed that only slow walking speed was significantly correlated with LTC after adjusting for all confounding factors except the MoCA-J score (HR = 1.74, 95% CI = 1.10–2.75, *P* = .018). However, slow walking speed was still a risk factor for LTC after adjusting for the MoCA-J score and other confounding factors (HR = 1.64, 95% CI = 1.03–2.60, *P* = .037). Moreover, other variables affecting the need for LTC included a lower MoCA-J score (HR = 0.93, 95% CI = 0.88–0.98, *P* = .005), an advanced age (80 years old, HR = 4.56, 95% CI = 2.55–8.16, *P* < .001; 90 years old, HR = 8.84, 95% CI = 3.81–20.52, *P* < .001; with 70 years old as a reference), and a female sex, with females showing a higher risk than males (HR = 1.63, 95% CI = 1.07–2.49, *P* = .023). In addition, on comparing cumulative hazards between the groups, both grip strength and walking speed are shown in Figs. 2 and 3. Moreover, adjusted Cox proportional hazard regression analyses following age stratification (only 70 and 80 years old) were implemented (Additional file 1: Table S1 & Additional file 2: Table S2), but the analysis of those in their 90s is not shown because no one in the normal walking speed group received LTC and the sample size was too small.

**Table 2:** Cox proportional hazard regression analyses of receiving LTC (*n* = 1098)

Variables	Model 1^a^		Model 2^b^		Model 3^c^	
	HR	95% CI	HR	95% CI	HR	95% CI
**Weak grip strength** ref. normal	2.12***	1.45–3.09	1.34	0.90–2.01	1.23	0.82–1.85
**Slow walking speed** ref. normal	2.24***	1.44–3.49	1.74*	1.10–2.75	1.64*	1.03–2.60
**MoCA-J score**	–	–	–	–	0.93**	0.88–0.98
**Age** (ref. = 70 y/o)						
80 y/o	–	–	5.11***	2.87–9.11	4.56***	2.55–8.16
90 y/o	–	–	11.35***	4.98–25.85	8.84***	3.81–20.52
**Sex** (ref. = male)	–	–	1.63*	1.07–2.49	1.63*	1.07–2.49
**HT**	–	–	1.01	0.61–1.68	0.99	0.60–1.64
**DM**	–	–	1.12	0.67–1.88	1.13	0.68–1.90
**Stroke**	–	–	1.41	0.64–3.08	1.44	0.66–3.15
**Joint diseases**	–	–	1.11	0.74–1.67	1.18	0.79–1.78
**Living alone**	–	–	1.34	0.86–2.10	1.42	0.91–2.23
**BMI**	–	–	1.01	0.94–1.08	1.00	0.93–1.08
**Serum albumin**	–	–	0.65	0.31–1.39	0.72	0.35–1.50

*BMI* body mass index, *CI* confidence interval, *DM* diabetes mellitus, *HR* hazard ratio, *HT* hypertension, *LTC* long-term care, *MoCA-J* The Japanese version of the Montreal Cognitive Assessment, *ref.* reference, *y/o* years old

*P < .05

**P < .01

***P < .001

^a^Model 1 was unadjusted for physical function (weak grip strength and slow walking speed)

^b^Model 2 was adjusted for physical function by age, sex, HT, DM, stroke, joint diseases, living alone, BMI, and serum albumin

^c^Model 3 was adjusted for physical function by MoCA-J score, age, sex, HT, DM, stroke, joint diseases, living alone, BMI, and serum albumin

## Discussion

The principal finding of the present study was that those receiving LTC among Japanese community-dwelling older and oldest people showed a significantly slower walking speed as an independent physical function variable. Additional predictors of receiving LTC were a lower MoCA-J score, an advanced age, and a female sex.

Long-term care was correlated with only a slow walking speed but was not significant for a weak grip strength, and this finding agreed with that of Chen et al. [6] who investigated the use of walking speed and grip strength to predict the cause of mortality and found that walking speed function impairment was a more powerful predictor of all-cause mortality at 3 years than grip strength. The study by Makizako et al. [16] also demonstrated a slower walking speed, expected following activity limitation even in independent older adults with no IADL limitation. This indicates that walking speed is worth considering as a predictive marker of IADL limitation. Walking speed was, therefore, superior to other physical performance tests, such as grip strength, in identifying populations at a higher risk of all-cause mortality. At the same time, the combination of walking speed with grip strength had no better prognostic benefit than using walking speed only in predicting mortality from all causes [6].

Walking speed is, according to Shinkai [17], a strong predictor of IADL impairment in older people. This indicates that retaining the walking capacity while living independently is important for older adults. Owing to the decrease in walking speed and IADL, older people often continue to lose out on opportunities to take part in recreational activities and other social life aspects. For older people, whether they need some support or are completely independent, maintaining that level of independence requires strategies such as maintaining the functional ADL and remaining socially active through regular leisure activities that promote maintenance of the walking capacity [18]. Moreover, a slow walking speed was recognized as a better predictor of cumulative deficits over multiple physiological systems, which may also be more useful in identifying older adults with higher health risk profiles and the cause of the need for LTC. From this point of view, a slow walking speed has had a significant effect on the need for LTC certification compared with weak grip strength, as walking speed is a complex activity requiring sophisticated coordination of multiple body systems [19] as well as adequate coordination to maintain strength production, structural support, movement, and balance control [20].

The need for LTC is related to not only independent factors such as a slow walking speed but also other variables such as a lower MoCA-J score, an advanced age, and a female sex. In the study of Nishiguchi et al. [21], they found that cognitive decline is an independent predictor of new LTC requirement certifications, and the severity of the cognitive decline in the elderly is positively associated with LTC requirement certification in Japan. In an LTC setting, cognitive functioning may be a robust predictor, because dysfunction can impair one’s ability to complete ADLs, as well as one’s ability to engage in safety precautions (e.g., preventing falls). Thus, changes in cognitive functioning may possibly further impair the physical function [22].

Advanced age- and sex-related differences in walking speed also emerged. In the study of Wu et al. [23], determinants of the need for LTC among older Taiwanese adults were examined, and they found that advanced age was a predictive factor for receiving LTC. This basically illustrates that people who are older usually show a decrease in health, and this, in effect, leads to the increased use of health care like LTC services [24]. While sex-related slow walking speed in females was a higher risk of the need for LTC compared with males. This phenomenon can be explained by women showing impaired exercise performance in the domains of lower extremity strength, balance, and ambulation, which means that a sedentary lifestyle will cause low social activity and then ADL or IADL limitations; subsequently, LTC needs among older people can increase [25].

In the present study, several limitations have to be mentioned. First, the participants in the study are not a representative sample of the general population of older Japanese people but rather were volunteers and, thus, possibly more health conscious. As such, selection bias might be inevitable in the present study. Second, our study population was limited, including only non-institutionalized, community-dwelling, age-homogeneous Japanese citizens in restricted areas, most of whom were mentally healthy and without dementia despite the sample being taken from a basic residential registry. Lastly, the number of people with a need for LTC may have been underestimated because this was measured using LTC insurance in the present study. People with such a need may thus have been grouped as not requiring LTC. Moreover, LTC data were collected by participants’ self-report. Information from national LTC database system may have more accuracy.

## Conclusion

The findings of this study may contribute to a better understanding of factors related to LTC. In particular, walking speed could be a useful indicator to classify older people at high risk of requiring LTC, which may be a criterion for disability prevention interventions and could serve as an outcome measure for physical function studies in older people.

## Supplementary information

**Additional file 1: Table S1.** Cox proportional hazard regression analyses of those receiving LTC (n = 523) (70 years old).

**Additional file 2: Table S2.** Cox proportional hazard regression analyses of those receiving LTC (n = 531) (80 years old).

## Data Availability

The datasets used and/or analyzed during the current study are available from the corresponding author on reasonable request.

## Abbreviations

ADL: Activities of daily living
BMI: Body mass index
BP: Blood pressure
CHS: Cardiovascular Health Study
CI: Confidence interval
DM: Diabetes mellitus
HR: Hazard ratio
HT: Hypertension
IADL: Instrumental activities of daily living
J-CHS: The Japanese version of Cardiovascular Health Study
LTC: Long-term care
MoCA-J: The Japanese version of the Montreal Cognitive Assessment
SONIC: The Septuagenarians, Octogenarians, Nonagenarians Investigation with Centenarians study
