# Supplementary material for: Association between physical function and long-term care in community-dwelling older and oldest people: the SONIC study
Source: Environ Health Prev Med. 2020 Sep 1;25:46. doi: 10.1186/s12199-020-00884-3 (PMC7465312; doi:10.1186/s12199-020-00884-3)
Supplement: Supplementary file 1 — Additional file 1: Table S1. Cox proportional hazard regression analyses of those receiving LTC (n = 523) (70 years old). [file 12199_2020_884_MOESM1_ESM.pdf]

Additional file 1: Table S1. Cox proportional hazard regression analyses of those receiving LTC (n=523) **(70 years old)**

Variables	Model 1[a]		Model 2[b]		Model 3[c]	
	HR	95% CI	HR	95% CI	HR	95% CI
**Weak grip strength** ref. normal	0.96	0.31-3.01	0.81	0.25-2.67	0.76	0.23-2.54
**Slow walking speed** ref. normal	1.25	0.44-3.52	1.13	0.38-3.34	1.12	0.38-3.30
**MoCA-J score**	-	-	-	-	0.89	0.76-1.06
**Sex** (ref. = male)	-	-	1.38	0.44-4.31	1.48	0.47-4.66
**HT**	-	-	0.97	0.31-3.11	0.92	0.29-2.93
**DM**	-	-	1.76	0.54-5.79	1.82	0.56-5.92
**Stroke**	-	-	1.81	0.23-14.56	2.04	0.26-16.34
**Joint diseases**	-	-	2.24	0.74-6.79	1.97	0.64-6.07
**Living alone**	-	-	1.96	0.60-6.44	2.09	0.63-6.92
**BMI**	-	-	1.11	0.95-1.30	1.10	0.95-1.29
**Serum albumin**	-	-	0.49	0.07-3.63	0.57	0.08-4.20

Abbreviations: BMI, Body mass index; CI, Confidence interval; DM, Diabetes mellitus; HR, Hazard ratio; HT, Hypertension; LTC, Long-term care; MoCA-J, The Japanese version of the Montreal Cognitive Assessment; ref., reference.

[a] **Model 1** was unadjusted for physical function (weak grip strength and slow walking speed).

[b] **Model 2** was adjusted for physical function by age, sex, HT, DM, stroke, joint diseases, living alone, BMI, and serum albumin**.**

[c] **Model 3** was adjusted for physical function by MoCA-J score, age, sex, HT, DM, stroke, joint diseases, living alone, BMI, and serum albumin.
