# Supplementary material for: Association between physical function and long-term care in community-dwelling older and oldest people: the SONIC study
Source: Environ Health Prev Med. 2020 Sep 1;25:46. doi: 10.1186/s12199-020-00884-3 (PMC7465312; doi:10.1186/s12199-020-00884-3)
Supplement: Supplementary file 2 — Additional file 2: Table S2. Cox proportional hazard regression analyses of those receiving LTC (n = 531) (80 years old). [file 12199_2020_884_MOESM2_ESM.pdf]

Additional file 2: Table S2. Cox proportional hazard regression analyses of those receiving LTC (n=531) **(80 years old)**

Variables	Model 1[a]		Model 2[b]		Model 3[c]	
	HR	95% CI	HR	95% CI	HR	95% CI
**Weak grip strength**	2.12**	1.35-3.32	1.76*	1.10-2.80	1.59	0.99-2.57
ref. normal						
**Slow walking speed**	2.00**	1.20-3.32	1.77*	1.05-2.99	1.65	0.97-2.80
ref. normal						
**MoCA-J score**	-	-	-	-	0.93*	0.88-0.99
**Sex** (ref. = male)	-	-	1.67*	1.00-2.77	1.67*	1.00-2.77
**HT**	-	-	0.96	0.53-1.75	0.98	0.54-1.79
**DM**	-	-	1.09	0.58-2.07	0.93	0.49-1.76
**Stroke**	-	-	1.48	0.59-3.75	1.48	0.59-3.75
**Joint diseases**	-	-	1.05	0.66-1.68	1.12	0.70-1.79
**Living alone**	-	-	1.36	0.81-2.29	1.44	0.85-2.42
**BMI**	-	-	0.99	0.91-1.08	0.98	0.90-1.07
**Serum albumin**	-	-	0.67	0.28-1.61	0.72	0.30-1.73

Abbreviations: BMI, Body mass index; CI, Confidence interval; DM, Diabetes mellitus; HR, Hazard ratio; HT, Hypertension; LTC, Long-term care; MoCA-J, The Japanese version of the Montreal Cognitive Assessment; ref., reference.

* $P < .05$ ** $P < .01$

[a] **Model 1** was unadjusted for physical function (weak grip strength and slow walking speed).

[b] **Model 2** was adjusted for physical function by age, sex, HT, DM, stroke, joint diseases, living alone, BMI, and serum albumin.

[c] **Model 3** was adjusted for physical function by MoCA-J score, age, sex, HT, DM, stroke, joint diseases, living alone, BMI, and serum albumin.
